# MHDA-Functionalized Multiwall Carbon Nanotubes for detecting non-aromatic VOCs

**DOI:** 10.1038/srep35130

**Published:** 2016-10-10

**Authors:** Atef Thamri, Hamdi Baccar, Claudia Struzzi, Carla Bittencourt, Adnane Abdelghani, Eduard Llobet

**Affiliations:** 1 Carthage University, National Institute of Applied Science and Technology, Bp676, 1080 Charguia Cedex, Tunisa, Tunisia; 2 Plasma-Surface Interaction Chemistry University of Mons, 1 Copernic, 7000 Mons, Belgium; 3 MINOS-EMaS, Universitat Rovira i Virgili, Avda. Països Catalans, 26, 43007 Tarragona, Spain

## Abstract

The chemical modification of multiwalled carbon nanotubes (MWCNTs) with a long chain mercapto acid is reported as a way to improve sensitivity and response time of gas sensors for detecting alcohols, acetone and toxic gases such as DMMP. We have developed sensors employing MWCNTs decorated with gold nanoparticles and modified with a 16-mercaptohexadecanoic acid (MHDA) monolayer. Morphological and compositional analysis by Transmission Electron Microscopy (TEM), Fourier Transform Infra-red Spectroscopy (FTIR) and X-ray photoelectron spectroscopy were performed to characterize the gold nanoparticles and to check the bonding of the thiol monolayer. The detection of aromatic and non-aromatic volatiles and DMMP vapors by MWCNT/Au and MWCNT/Au/MHDA shows that the presence of the self-assembled layer increases sensitivity and selectivity towards non-aromatics. Furthermore, it ameliorates response dynamics, and significantly reduces nitrogen dioxide and moisture cross-sensitivity.

The monitoring of the environment requires devices that must be fast, sensitive, stable and selective to detect the pollutants and toxic gases/vapors in a simple and efficient way^1^,^2^,^3^. Since 1962, when it was reported that the interaction between gas molecules and the surface of a metal oxide semiconductor altered the conductivity of the material, the advances in manufacturing techniques have enabled the production of low cost sensors with improved sensitivity^4^,^5^,^6^,^7^,^8^. Additionally, also gas sensors employing organic semiconductor materials have been reported for the detection of hazardous gases in air, and especially, organic pollutants^9^,^10^. More recently, Kong *et al*. first explored in ref. 11 the use of carbon nanotubes as gas sensitive nanomaterials, given their remarkable intrinsic properties such as high surface area to volume ratio, high carrier mobility, high physicochemical stability and high adsorption capacity^12^,^13^,^14^. Mats of CNTs are well known to behave generally as mild *p*-type semiconductors and respond to both oxidizing and reducing gases under ambient conditions^11^,^12^,^13^,^14^. However, gas sensors based on pristine CNTs exhibit some disadvantages such as high inertness and, therefore, low interaction with their chemical environment. This results in very low sensitivity to many relevant gases^15^. Additionally, very oxidizing gases such as NO_2_ or O_3_ strongly bind to carbon nanotubes, resulting in extremely long sensor recovery dynamics or even in partially irreversible responses^16^. To overcome these drawbacks, the physical and chemical modification of CNTs has been implemented, especially by substitutional doping^17^,^18^,^19^, by grafting functional groups to the outer wall of tubes via wet chemistry or reactive plasma routes^15^,^20^,^21^ and/or by decorating its surface employing a wide range of materials such as metal oxide nanoparticles^22^,^23^,^24^,^25^,^26^, polymers^27^,^28^,^29^,^30^, non-polymeric organic materials^31^,^32^,^33^ or catalytic metal nanoparticles (such as Rh, Pd, Pt or Au)^16^,^34^,^35^,^36^,^37^,^38^,^39^,^40^,^41^,^42^. Once functionalized, carbon nanotubes have been found responsive to a wide spectrum of gaseous species including nitrogen dioxide, ozone, ammonia, warfare agents and both aromatic and non-aromatic volatile organic compounds^43^. Modified carbon nanotubes provide improvements in gas detection performance with enhanced response and selectivity at room temperature. Metal and metal oxide nanoparticles donate or accept a significant amount of charge upon gas adsorption, so as to affect carrier transport in the carbon nanotube^44^. Polymers or non-polymeric organic materials are often used to filter out polar or non-polar compounds, so better selectivity can be achieved and cross-sensitivity to ambient moisture is diminished^32^,^33^. Very recently, we have employed quinoxaline-walled thioether-legged deep cavitand functionalized multiwall carbon nanotubes for the detection of benzene vapors at trace levels^45^. The cavitand is grafted onto gold nanoparticle decorated multiwall carbon nanotubes by a self-assembled monolayer process.

In this paper we develop resistive gas sensors employing films of gold nanoparticle decorated, multiwalled carbon nanotubes (MWCNT/Au) functionalized with a self-assembled monolayer of 16 Mercaptohexadecanoic acid (MHDA), designated as MWCNT/Au/MHDA. The self-assembled monolayer (SAM) technique employed here takes advantage of the good affinity between the sulfur group (thiol) present at one end of the MHDA molecule and gold, which decorates the outer wall of carbon nanotubes in the form of nanoparticles. The SAM technique has been routinely employed for many years in the synthesis of active films for biosensor applications^46^,^47^. Transmission electron microscopy (TEM), Fourier transform Infrared (FTIR) spectroscopy and X-ray photoelectron spectroscopy (XPS) characterizations were carried out to analyze the chemical and structural composition of our MWCNT/Au/MHDA films and to check the success in the functionalization. The response of both MWCNT/Au and MWCNT/Au/MHDA sensors towards aromatic, non-aromatic volatiles and a warfare agent simulant (DMMP) is studied and, the role of MHDA in the sensing properties is discussed in light of the experimental findings.

## Experimental

### Synthesis, plasma treatment and gold decoration of carbon nanotubes

In this part the steps employed for the preparation of multiwall carbon nanotubes (MWCNT) are described. The carbon nanotubes used in the experiment were purchased from Nanocyl, S.A (Belgium). They were synthesized by catalytic chemical vapor deposition and their purity was higher than 95% (NanocylTM NC3100). These were up to 50 microns in length and their outer and inner diameters ranged from 3 to 15 nm and 3 to 7 nm, respectively^48^. In the first step, MWCNTs were treated in an oxygen plasma to create oxygenated surface defects^15^,^49^. This method consists of introducing the MWCNTs in the chamber of an ATC Orion-8-HV multi-target sputtering machine (AJA International, Inc., USA). An oxygen plasma treatment is then conducted at a pressure of 0.1 Torr, using a power of 15 W for 1 min. Inductively-coupled plasma at a RF frequency of 13.56 MHz is used during this process. A controlled flow of oxygen and argon is introduced inside the chamber, which results in functional oxygen species (mainly carbonyl, carboxyl and hydroxyl groups) being attached to the carbon nanotube sidewalls. These controlled oxygenated defects are known to act as nucleation sites for metal nanoparticles^21^. MWCNTs are subject to a mild oxygen plasma treatment that respects their integrity^16^,^21^. In the second step, the deposition of MWCNT films onto alumina sensor substrates is performed by airbrushing. Film resistance is monitored during deposition to achieve good device to device reproducibility. Films are thin and optically transparent. Full details on this procedure have been reported elsewhere^45^,^50^. Then the plasma treated MWCNT films were decorated with Au nanoparticles by sputtering method. The sputtering parameters were adjusted at 30 W, pressure was kept at 3.75 mTorr, a controlled flow of argon was introduced inside the chamber and the process duration was 10 s.

### Functionalization with long alkyl-thiols chains

Self-assembled monolayers (SAMs) consisting of long alkyl-thiols chains on metal surfaces have been shown to be stable in air, water and organic solvents at room temperature^51^.

**Figure 1:**
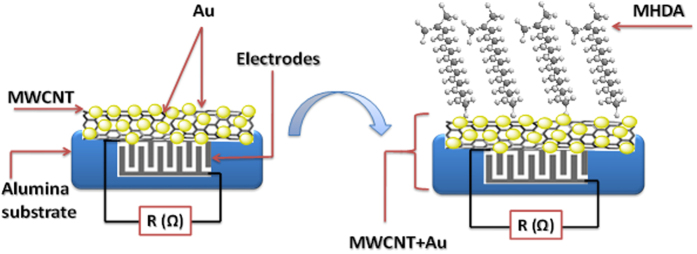
Synoptic sensor structure based on MHDA deposited on carbon nanotubes decorated with gold nanoparticles

Here the plasma treated, Au nanoparticle decorated MWCNTs were further functionalized by capping Au-nanoparticles with MHDA employing a SAM technique. A 2 ml drop of MHDA 0.1 mM dissolved in ethanol was deposited on the gold decorated multiwall carbons nanotube film kept at 4 °C, so the solvent took 12 hours to evaporate. Then, the substrates were rinsed extensively with ethanol and dried under a N_2_ flow. This procedure was repeated 4 times in order to remove unbound thiols^52^. Figure 1 shows the synoptic structure of the sensor before and after the deposition of MHDA on the MWCNT/Au sensor.

### TEM and XPS characterizations

The morphology and chemical composition of the plasma treated, metal decorated and MHDA functionalized multiwall carbon nanotube films were characterized using transmission electron microscopy and by X-ray photoelectron spectroscopy (XPS), respectively.

TEM analysis was carried out on a JEOL model 1011 operating at 100 kV. XPS, was employed as a way to confirm the presence of MHDA grafted to the surface of Au-decorated MWCNTs. A VERSAPROBE PHI 5000 spectrometer from Physical Electronics, equipped with a Monochromatic Al Kα X-ray source was used. The energy resolution was 0.6 eV. For the compensation of built-up charge on the sample surface during the measurements, a dual beam charge neutralization composed of an electron gun (≈1 eV) and an Ar ion gun (≤10 eV) was used, as reported in ref. 53.

### FT-IR characterization

To further confirm the immobilization of MHDA on the MWCNT/Au films, an alpha FT-IR spectrometer (Bruker, France) equipped with a platinum ATR single reflection diamond-sampling module (Bruker Optics) was used. Infrared spectra were collected at an average of 24 scans per sample between the wave number range of 4000–450 cm^−1^ at a resolution of 4 cm^−1^, and the Optics User Software (OPUS) version 6.5 (Bruker Optics) was used to analyze spectra. The analysis was kept simple since at first the spectrum of a bare substrate was recorded and considered as a reference. In the second step, the spectrum of a substrate having a Au-decorated MWCNT film was recorded and finally, the spectrum of an MHDA-functionalized, gold decorated MWCNT film was recorded. The difference between a given sample spectrum and the reference spectrum (i.e., bare substrate) were considered to study the presence of the MHDA monolayer^54^.

### Gas sensing measurements

The different sensors produced were tested for the detection of vapors from non-aromatic (ethanol, methanol, and acetone) and aromatic (toluene, benzene) volatile organic compounds (VOCs). Additionally, sensors were tested also with dimethyl-methyl-phosphonate (DMMP) a surrogate for the warfare agent SARIN. Pure dry air was used as balance/carrier gas.

Aromatic and non-aromatic VOCs were tested at INSAT (Tunisia). A pure dry air cylinder (Air Liquide) was connected to two mass flow meters and a thermostated bubbler to generate reproducible concentrations of the various VOCs tested. These were coupled to a stainless steel sensor chamber (35 cm^3^ volume) in which up to three sensors could be connected^26^,^42^,^50^. The flow was set to 200 sccm. An HP 4192A impedance analyzer was used to perform the measurements. These measurements were conducted employing the impedance analyzer operating at a fixed frequency of 1 kHz, since all sensors showed a purely resistive behavior below 100 kHz.

DMMP was measured at URV lab. (Spain). An automated mass-flow system, which used pure dry air (AIR PRODUCTS) and a calibrated permeation tube was employed to generate reproducible concentrations of DMMP vapors. Humidity measurements were performed using an Environics series 4000, which allowed us to generate humidified gas mixtures. The sensors characterized were those also studied in Tunisia and the test chamber and the gas flow were identical to those used in Tunisia. The resistance of the sensors was measured with an Agilent 34972A multimeter.

For all the measurements performed, sensor response is defined as the normalized resistance variation, which can be expressed as follows:


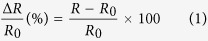


where *R* is the resistance of a sensor measured in the presence of a pollutant, and *R*_0_ is the sensor baseline resistance (i.e. when the sensor is in the presence of clean carrier gas).

## Results and discussion

### Transmission electron microscopy and XPS analysis

**Figure 2:**
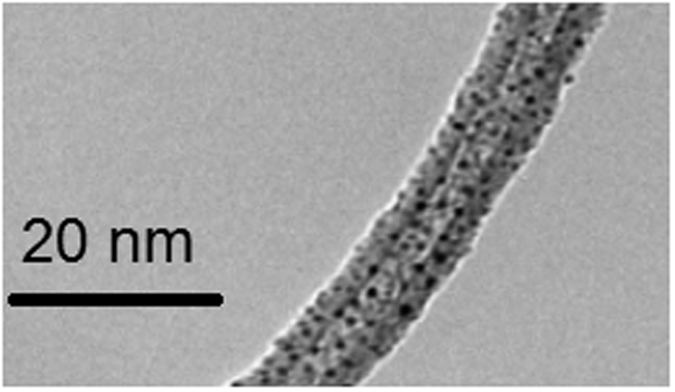
TEM micrograph of a Au-decorated MWCNT.

Samples of the Au-decorated MWCNT films were mechanically removed from the alumina sensor substrate and deposited on standard copper TEM grids. A standard resolution TEM micrograph is shown in Fig. 2. Au nanoparticles show a narrow, mono-modal diameter-size distribution with average value equal to 2 nm. TEM micrographs taken after conducting the SAM functionalization with MHDA do not show significant differences (e.g. particle coalescence is not observed), most probably due to the low temperature (i.e. 4 °C) employed to perform this process.

**Figure 3:**
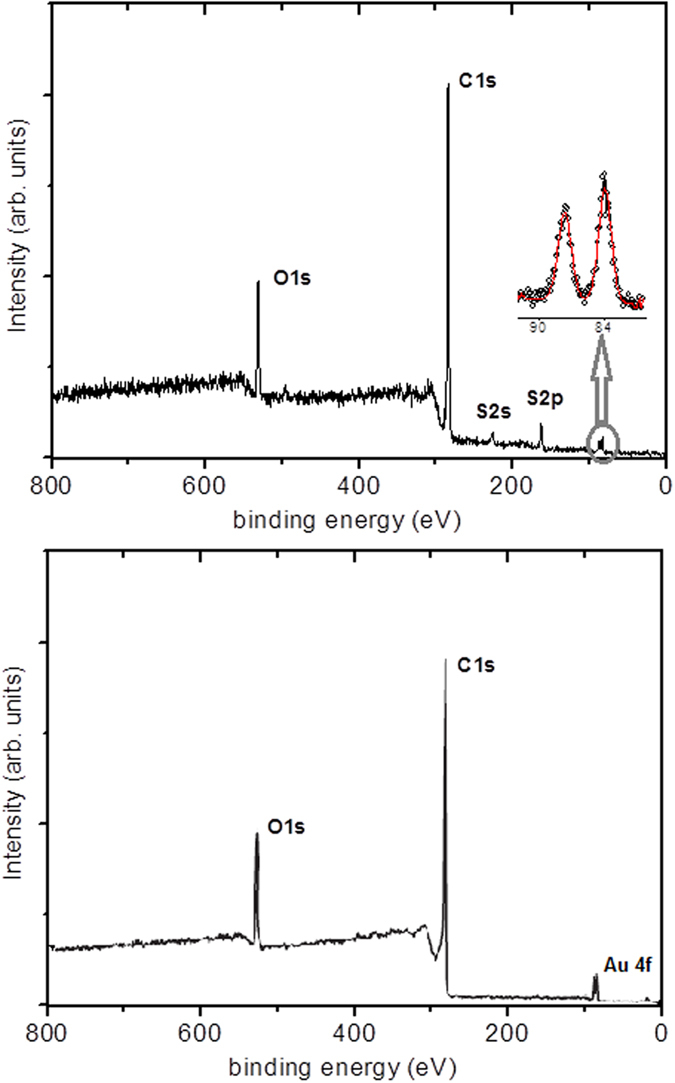
Survey spectra of the MWCNT/Au/MHDA (top) and MWCNT/Au (bottom) gas-sensitive nanomaterials.

The composition of oxygen plasma-treated MWCNT/Au and MWNT/Au/MHDA films has been studied in detail by employing XPS techniques. Figure 3 shows the XPS spectra recorded on MWNT/Au/MHDA and MWCNT/Au sensors. For both samples, the most intense structure peaking at 284.6 eV is characteristic of photoelectrons emitted from carbon atoms in sp2 bonding. The peak at 532 eV corresponds to photoelectrons emitted from oxygen and results mainly from the oxygen plasma treatment undergone by CNTs prior to Au decoration. The peak at 162.9 eV, which appears exclusively in the MWCNT/Au/MHDA sample, corresponds to sulfur atoms testifying the presence of the 16-Mercaptohexadecanoic acid grafted at the CNT surface. The inset in Fig. 3 (top) shows the doublet peaks due to photoemission from Au atoms at 84 eV (4 f7/2) and 87.6 eV (4 f5/2). The presence of these doublets in the two samples analyzed, indicates that the metal sputtering parameters were optimal for the metal decoration of the CNTs. Table 1 summarizes the relative atomic composition of the MWCNT/Au and MWCNT/Au/MHDA films, derived from the XPS analysis.

The decrease in the relative intensity of the Au doublet observed for the MHDA functionalized sample is due to the shielding effect of the compact MHDA layer grafted to the surface of CNTs. The S/Au atomic ratio (considering the %Au in the MWCNT/Au sample and the %S in the MWCNT/Au/MHDA sample) suggests the formation of a monolayer. This is further supported by SEM inspection of the MWCNT/Au/MHDA film, which can be found in the Supporting Information.

### FT-IR analysis

**Figure 4:**
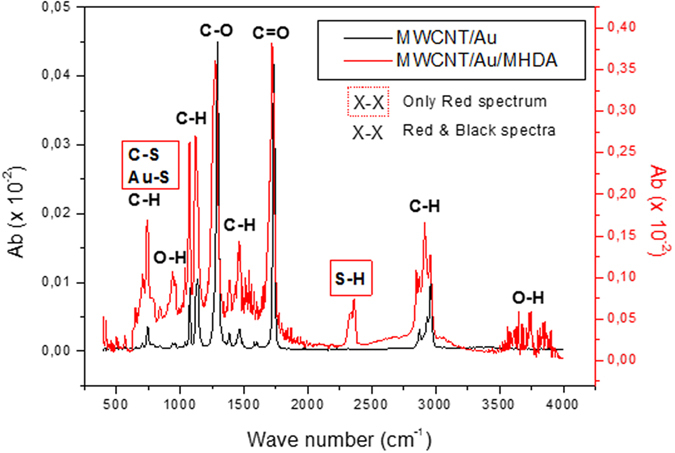
FT-IR spectra of gold nanoparticle decorated MWCNTs and 16-mercaptohexadecanoic acid deposited on MWCNs/Au samples.

Figure 4 compares the FT-IR spectra of MWCNT/Au and MWCNT/Au/MHDA samples. Both spectra show six bands appearing at 3630, 2900, 1700, 1450, 1280 and 1056 cm^−1^, which can be assigned to O–H stretching, C–H stretching, C = O stretching, C–H bending, C– O stretching and C–H anti-stretching vibration mode, respectively^55^. These bands correspond to the oxygenated defects present at the surface of MWCNTs, to the carboxylic groups and the C-H bonds present in both samples. The formation of Au–S and C–S bonds present in the MHDA functionalized sample is proven by the substantial increase in absorbance intensity in the 685 to 790 cm^−1^ band^56^, in comparison to the small absorbance intensity recorded for the MWCNT/Au sample in this region. In particular, the Au-S stretch vibration mode peaks at 760 cm^−1 ^56. The 685 to 790 cm^−1^ band appears in both samples, because it can be also assigned to the stretch mode of C–H groups present in MHDA, to the sp2 C–H bond present in MWCNTs and to the O–H deformation mode present in both materials. Finally, another band appears centered at 2300 cm^−1^ in the MHDA-functionalized sample exclusively, which corresponds to the S–H stretch vibration mode^57^. This is indicative that some non-linked MHDA remains on the surface, even after the cleaning procedure. However, the amount of non-linked MHDA is low (according to the S/Au ratio derived from the above-discussed XPS results.

In summary, both XPS and FTIR results confirm the covalent functionalization of Au-decorated MWCNTs with MHDA, although FTIR reveals that some residual, small amount of non-covalently bound MHDA remains on top of the SAM.

### Sensing results

#### Detection of aromatic and non-aromatic VOCs

**Figure 5:**
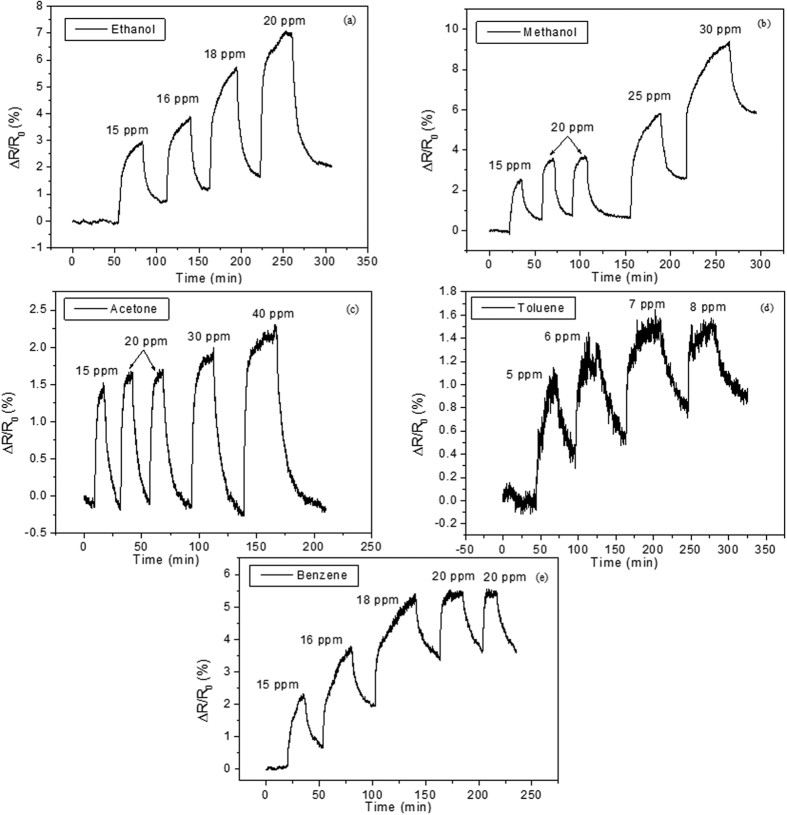
Response and recovery curves for a MWCNT/Au sensor in the presence of increasing concentrations of (**a**) ethanol, (**b**) methanol, (**c**) acetone, (**d**) toluene and (**e**) benzene vapors.

The detection of aromatic and non-aromatic vapors was studied for Au-decorated MWCNT and MHDA functionalized, Au-decorated MWCNT sensors operated at room temperature. Figure 5 shows the response and recovery curves for MWCNT/Au sensors. Sensor resistance increases for increasing concentrations of the species tested. For non-aromatic volatiles, the detection mechanism involves the adsorption of the species either on Au NPs or on the outer wall of carbon nanotubes with electronic charge being transferred towards the CNT, which results in a decrease in conductivity^16^,^44^. While for acetone the baseline resistance is regained during the cleaning phase, this is not the case for the remaining species tested and short-term drift appears. In the case of ethanol and methanol this could be addressed by extending the cleaning phase. For aromatics, the nature of the interaction is stronger and likely involves π-π interactions between the aromatic ring and the external wall of carbon nanotubes^44^. A saturation effect is visible in the response towards aromatic volatiles, because these molecules are not completely desorbed form the surface of the sensors at room temperature. The interaction with aromatic compounds shifts the Fermi level of the MWCNT away from the valence band, resulting in a decrease of MWCNT conductance^44^,^45^.

Unlike for MWCNT/Au sensors, MWCNT/Au/MHDA sensors did not respond to aromatic VOCs (see the Supporting Information). Given the homogeneous dispersion of Au nanoparticles on the surface of carbon nanotubes (see Fig. 2) the SAM of MHDA densely envelops carbon nanotubes, which prevents aromatic VOCs from reaching their surface.

The carboxylic group of the MHDA functionalized MWCNTs is not suitable for adsorbing aromatic VOCs such as toluene and benzene. Therefore, aromatics remain far away from the surface of MWCNTs^58^. Furthermore, the MHDA layer prevents the establishment of π-π interactions (between the aromatic ring and CNT) or the direct adsorption of the aromatic molecule on Au NPs, which results in these species remaining largely undetected. High sensitivity to aromatic VOCs such as benzene or toluene has been reported for NWCNTs functionalized with calix^4^arenes^45^,^58^. These have a highly polarizable pocket and are very effective at adsorbing aromatics.

**Figure 6:**
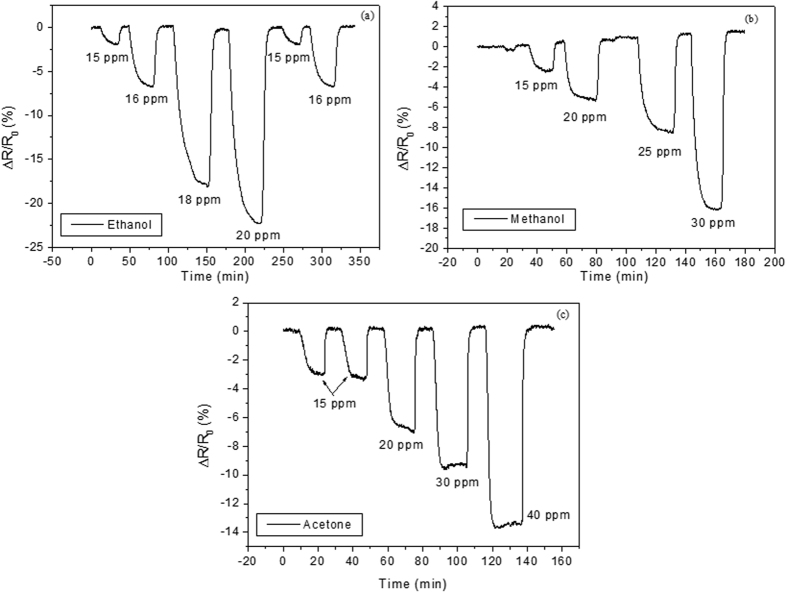
Response and recovery curves for a MWCNT/Au/MHDA sensor in the presence of increasing concentrations of (**a**) ethanol, (**b**) methanol, (**c**) acetone vapors.

On the other hand, the MWCNT/Au/MHDA sensor shows strong and fully reversible responses to the non-aromatic VOCs tested (see Fig. 6). The nice reversibility combined with fast response kinetics suggests that non-aromatics are physisorbed on the SAM of MHDA. MHDA is a long-chain mercaptan (longer than 6 carbon atoms) with a hydrophilic carboxylic acid tail, known to be soluble in alcohols and acetone, therefore affinity exists between MHDA and the non-aromatic VOCs used. The presence of hydroxyl and carboxyl functional groups in CNTs has been reported to favor interactions via hydrogen bond with hydrogen accepting molecules such as acetone or dipole-dipole with polar molecules such as the tested alcohols^58^. Furthermore, polyaniline functionalized with thiols having hydrophilic functional groups such as carboxyl shows an enhanced sensitivity to alcohols, which increases with the length of their carbon chain^59^. Therefore, the carboxyl group of the MHDA molecule is responsible for the remarkable response of the MWCNT/Au/MHDA film towards ethanol, methanol or acetone. One important feature is that the sign of the response is reversed with respect to that of the MWCNT/Au sensor (i.e. resistance diminishes for increasing concentrations of non-aromatic VOCs). Upon adsorption of the non-aromatic VOCs electronic charge is transferred from CNTs towards the carboxylic group on the surface and a decrease in sensor resistance is observed. This is due to the establishment of dipole-dipole interactions between the gas-sensitive mat and the physisorbed, polar, non-aromatic VOCs tested^60^. This slightly perturbs the band structure of MWCNTs causing a small shift of the Fermi level energy toward lower energies, which is equivalent to a *p*-doping of the tubes and their conductivity is increased^44^.

**Figure 7:**
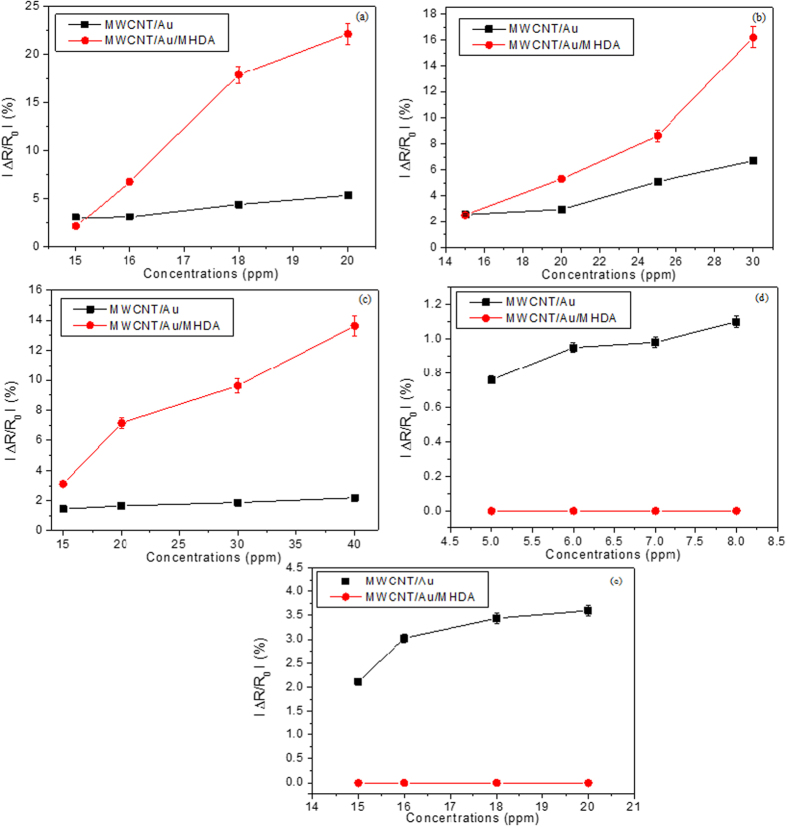
Calibration curves for the MWCNT/Au and MWCNT/Au/MHDA sensors for the detection of (**a**) ethanol, (**b**) methanol, (**c**) acetone, (**d**) toluene and (**e**) benzene vapors

The calibration curves for the different species tested and the two types of sensors studied are shown in Fig. 7. The results show that the sensitivity (slope of the calibration curves) towards alcohols and acetone of the sensor that comprises a SAM of MHDA is better that the bare sensor. Additionally the MHDA functionalized sensor is not responsive to the aromatic VOCs tested.

The sensitivity of the two types of sensors was calculated from the slope of the calibration curves (see Table 2). Sensors employing the MHDA functionalized film show a 3 to 11-fold increase in sensitivity towards alcohols and acetone. In these sensors the response to aromatic VOCS is suppressed.

The reason for the difference in response intensity between benzene and toluene vapors in MWCNT/Au may be related to the modification of the adsorption capacity of CNTs brought about by the decoration with metal NPs. It has been shown, as illustrated in Table 3, that MWCNTs can be made more responsive to either benzene or toluene, by selecting the metal employed for decorating MWCNTs.

MWCNT/Au/MHDA sensors were tested against ethanol after having undergone two months of storage either under ambient or under dry nitrogen conditions. The results reveal that after this period of elapsed time, sensor response remains largely unchanged for sensors stored under inert conditions and diminishes about 30% for sensors stored at ambient conditions, probably due to oxidation.

### Response time

**Figure 8:**
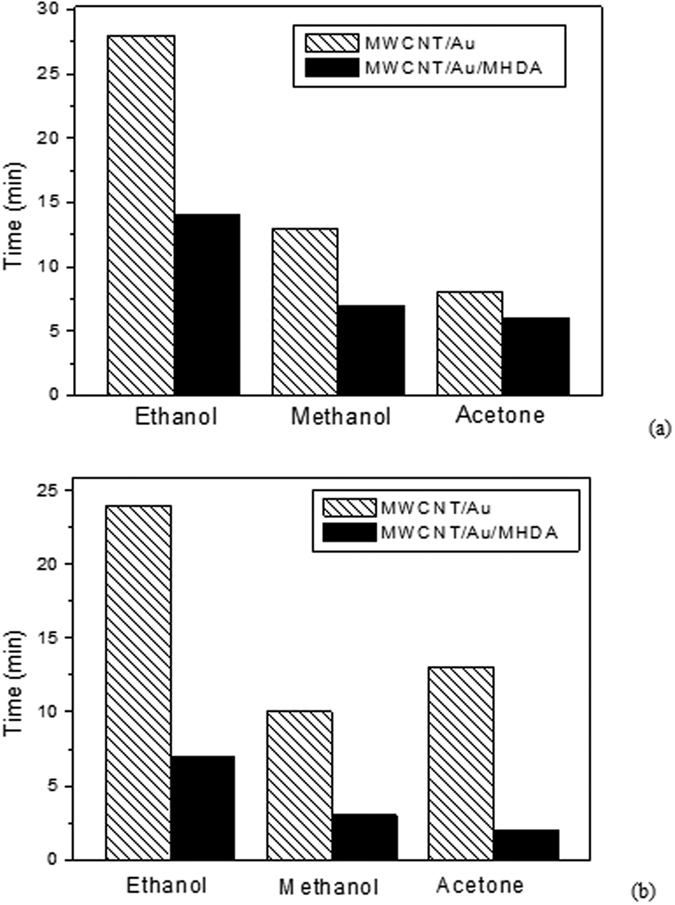
(**a**) Response time, (**b**) Recovery time of MWCNT/Au and MWCNT/Au/MHDA sensors in the presence of 15 ppm of ethanol, methanol or acetone vapors.

The response and recovery times were estimated for the two types of sensors studied and the different volatiles measured. Response (recovery) time is defined as the time needed for reaching 90% of the maximum response change (recovery change towards the baseline). Results are summarized in Fig. 8.

The results show that the rise and fall time of the sensor functionalized with thiol is better than for the Au-decorated MWCNTs sensors. This can be explained by the fact that in MHDA functionalized nanotubes, only weak physisorption interactions with VOCs take place, and these are characterized by fast kinetics. On the other hand, in non-functionalized nanotubes (i.e. MWCNT/Au), both stronger binding between VOCs and surface defects generated on the outer wall of CNTs by the oxygen plasma treatment and direct adsorption of VOCs on Au NPs occurs^51^, which explains the slower response and recovery kinetics. This is also the case for MWCNT/Au sensors detecting aromatic volatiles in which stronger non-covalent interactions (i.e., π-π) exist between the aromatic ring of the VOCs tested and the carbon nanotube walls^61^. As a result, response and recovery kinetics for aromatic VOCs are even slower than those reported in Fig. 8 for non-aromatic VOCs.

To discuss further the response and recovery times of the different sensors in terms of analyte sorption considering thermodynamics, the effect of the sensor operating temperature was studied. In addition to room temperature operation, sensors were kept heated at 120 °C and ethanol vapors were measured. These results are summarized in the supporting information. For both sensors, response intensity dramatically decreases with operation temperature (by more than a factor of two), while response dynamics are slightly improved. This is in agreement with previously reported results^16^,^19^. The fact that response and, thus, analyte adsorption decreased with temperature, is indicative of the exothermic nature of the sorption process^62^.

### Cross-sensitivity to nitrogen dioxide and ambient moisture

It is well-know that Au-decorated CNT sensors are highly responsive to nitrogen dioxide^44^. Experimental and *ab-initio* results have shown that the detection of nitrogen dioxide is further enhanced by the presence of carbonyl and carboxyl groups (i.e. oxygenated defects) on the surface of CNTs^15^. The response of MWCNT/Au and MWCNT/Au/MHDA sensors to 100 ppb and 500 ppb of nitrogen dioxide was investigated. The response of MWCNT/Au/MHDA sensors to NO_2_ was found to be about 30 to 50 times lower than that of MWCNT/Au sensors (see the Supporting Information). According to ab-initio computations, NO_2_ molecules bind to carboxylic groups^15^,^44^. Therefore, nitrogen dioxide molecules are attracted by the carboxylic acid tail of MHDA, and remain far away from the surface of MWCNTs. This binding does not generate a sensor signal because the long chain of the mercaptan prevents charge transfer between the molecule and the CNT. However, NO_2_ molecules are small, and some reach the surface of and interact with CNTs, which explains the response observed. The sensitivity towards NO_2_ is 45·10^−2^ % × ppm^−1^, which is about 10 times lower than the sensitivity towards ethanol and similar to the one for acetone (see Table 2). On the other hand, for MWCNT/Au sensors (i.e., sensors in which CNTs are not coated with MHDA), the sensitivity towards NO_2_ is significantly higher (3750·10^−2^ % × ppm^−1^).

The response of the sensors to changes in the moisture content was studied (these results are summarized in the Supporting Information). MWCNT/Au sensors were more affected than MWCNT/Au/MHDA sensors by changes in the humidity background. The hydrophilic nature of the carboxyl terminal present in MWCNT/Au/MHDA binds the water molecule far away from the CNTs. The baseline resistance of this sensor is not heavily affected when the relative humidity changes between 50% and 80%. This is possibly due to the formation, at humidity levels below 50%, of a monolayer of water molecules, adsorbed at the hydrophilic terminals of the MHDA molecules grafted to CNTs. To further study the effect of moisture on sensor response, the detection of 20 ppm of ethanol in air humidified at 50% R.H was measured. While the response of bare MWCNT/Au sensors is about 50% of the response in dry air, the response of MWCNT/Au/MHDA is about 93% of the response in dry air. These results are summarized in Table 3, which shows also the sensing properties of previously reported, differently functionalized MWCNTs.

The MHDA functionalization is beneficial because it boosts sensitivity to non-aromatic VOCs, suppresses the response towards aromatic VOCs such as benzene and toluene and keeps relatively low the cross-sensitivity to nitrogen dioxide. Furthermore, the sensitivity towards non-aromatic VOCs (e.g. ethanol) is remarkably resilient to the presence of humidity in the background.

### DMMP detection

**Figure 9:**
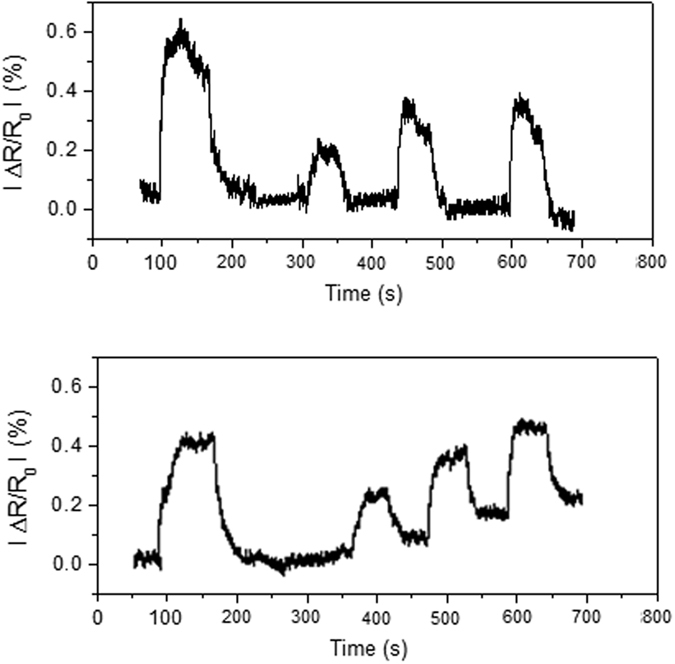
Response and recovery curves of a MHDA-Au-MWCNT sensor (top) and a Au-MWCNT sensor (bottom) towards 7, 1, 3.5 and 3.5 ppm of DMMP diluted in dry air.

The detection of DMMP vapors diluted in air was studied. Figure 9 shows the response and recovery curves for MWCNT/Au/MHDA and for MWCNT/Au sensors. Sensor resistance increases for increasing concentrations of DMMP in air. The detection mechanism involves a physisorption phenomenon for the MWCNT/Au/MHDA sensor characterized by fast response kinetics and absence of baseline drift. A stronger binding between DMMP and the surface of the MWCNT/Au sensor takes place because the presence of baseline drift can be observed, together with slower response kinetics.

**Figure 10:**
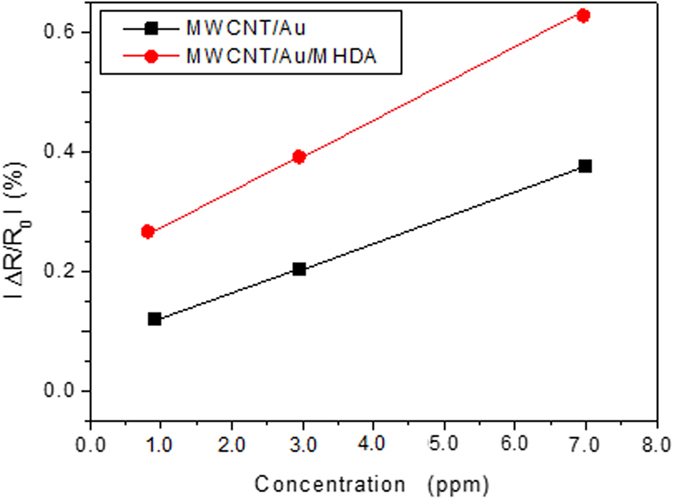
Calibration curves of MWCNT/Au and MWCNT/Au/MHDA sensors for the detection of DMMP vapors diluted in air.

The comparison of the response of the two types of sensors towards different concentrations of DMMP diluted in air is presented in Fig. 10. The results show that a better response and sensitivity (i.e., slope of the curve) is obtained for the MWCNT/Au/MHDA sensor. Given the response signal at 1 ppm and the noise level, the limit of detection for DMMP is in the hundreds of ppb.

The DMMP molecule has a highly polarized P = O bond and previous studies reported that this molecule has a dipole moment that ranges between 3 and 3.62 D^64^. The DMMP molecule is a hydrogen bond acceptor and thus can adsorb on the carboxyl group of the MHDA. The high electronegativity of the phosphonate group could explain that, upon adsorption, the Fermi level of the MWCNT is shifted away from the valence band, resulting in a decrease in the film conductance as observed experimentally.

## Conclusions

The self-assembly technique is a fast and simple method for the formation of an ultrathin film on top of gold nanoparticle-decorated mutiwalled carbon nanotubes. The sulfur moiety has proved to be an excellent anchor group on gold surface and the SAM of 16-mercaptohexadecanoic acid provides a convenient means for tuning gas sensitivity and selectivity of carbon nanotubes. In particular, thiol functionalized carbon nanotubes become insensitive to aromatic VOCs and significantly increase their response to non-aromatic VOCs such as alcohols and acetone. This behavior can be attributed to the carboxylic group in MHDA, which is suited for interacting with ketones via hydrogen bonding and polar alcohol molecules via dipole-dipole interaction. Furthermore, this functionalization results in a remarkable de-sensitization of the resulting nanomaterial to nitrogen dioxide and moisture. The functionalization also increases response and sensitivity towards organophosphorus compounds such as DMMP (once more, a hydrogen bond acceptor molecule that adsorbs at the carboxyl groups of the MHDA functionalized CNTs). This strategy opens a simple and flexible way to fine tune the gas reactivity of carbon nanotubes, since the length of the mercaptan chain and/or the hydrophilic or hydrophobic nature of the terminal part of the molecule become parameters of the functionalization. This strategy will be further studied in the near future.

## Additional Information

**How to cite this article**: Thamri, A. *et al*. MHDA-Functionalized Multiwall Carbon Nanotubes for detecting non-aromatic VOCs. *Sci. Rep*. **6**, 35130; doi: 10.1038/srep35130 (2016).

## Supplementary Material

Supplementary Information

## Figures and Tables

**Table 1:** Elemental chemical composition as derived from an XPS analysis of the MWCNT/Au and MWCNT/Au/MHDA samples.

elements	C1s	O1s	Au4f	S2p
MWCNT/Au	82.3%	15.2%	2.5%	—
MWCNT/Au/MHDA	82.1%	14.3%	0.3%	3.3%

**Table 2:** Mean values of the sensitivity (10^−2^ % × ppm^−1^) to the different vapors tested for the MWCNT/Au and MWCNT/Au/MHDA sensors.

	Ethanol	Methanol	Acetone	Toluene	Benzene
MWCNT/Au	45.6	37.3	2.8	11	27.8
MWCNT/Au/MHDA	525.3	108.9	32.4	N/D	N/D

N/D: Not detected

**Table 3:** Sensitivity (×10^−2^ % · ppm^−1^) of differently functionalized MWCNT sensors to VOCs and nitrogen dioxide.

	Benzene	Toluene	Methanol	Ethanol	Acetone	Nitrogen dioxide	Ref.
MWCNT/Au	27.8	11.7	37.3	45.6	2.8	3750	TW
MWCNT/Au/MHDA	N/D	N/D	108.9	523.3 (488.5 @ 50% R.H.)	32.4	45	TW
MWCNT/Au/Cav	420 (48 @ 60% R.H.)	11	N/A	17	N/A	430	45
MWCNT/Pt	N/A	N/A	6.7	9.9	2.1	9.4	16, 42
MWCNT/Pd	3.5	N/A	6.7	9.9	1.9	6.9	16, 42
MWCNT/Rh	3.3	28	12.5	7.7	2.5	5.5	41, 63
MWCNT/FeO	25	58	5	26	0.8	N/A	26

Measurements were performed under dry conditions unless otherwise specified.

TW: This Work; N/D: Not Detected; N/A: Not available.
