# Supplementary material for: MHDA-Functionalized Multiwall Carbon Nanotubes for detecting non-aromatic VOCs
Source: Sci Rep. 2016 Oct 10;6:35130. doi: 10.1038/srep35130 (PMC5056342; doi:10.1038/srep35130)
Supplement: Supplementary Information [file srep35130-s1.pdf]

Supporting information for:

MHDA-Functionalized Multiwall Carbon Nanotubes for detecting non-aromatic VOCs

Atef Thamri[1*], Hamdi Baccar[1], Claudia Struzzi[2], Carla Bittencourt[2], Adnane Abdelghani[1], Eduard Llobet[3*]

[1]Carthage University, National Institute of Applied Science and Technology, Bp676, 1080 Charguia Cedex, Tunisa, Tunisia

[2]Plasma-Surface Interaction Chemistry University of Mons, 1 Copernic, 7000 Mons, Belgium

[3]MINOS-EMaS, Universitat Rovira i Virgili, Avda. Països Catalans, 26, 43007 Tarragona, Spain

Corresponding authors: atef.th@hotmail.fr, eduard.llobet@urv.cat

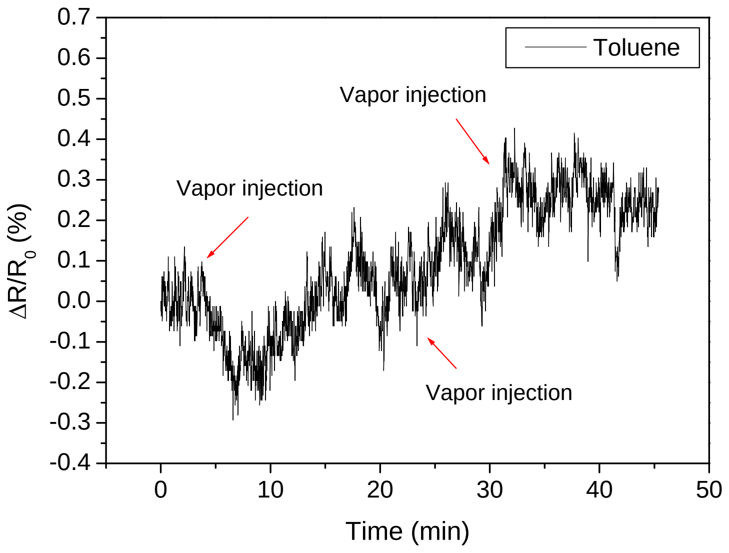

(a)

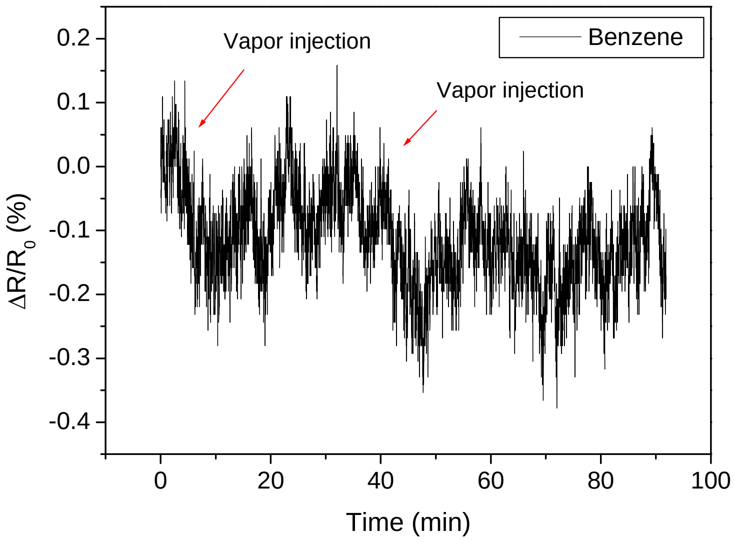

(b)

Figure S.1: Response curves of detection by MWCNT/Au/MHDA sensor of (a) toluene and (b) benzene vapors.

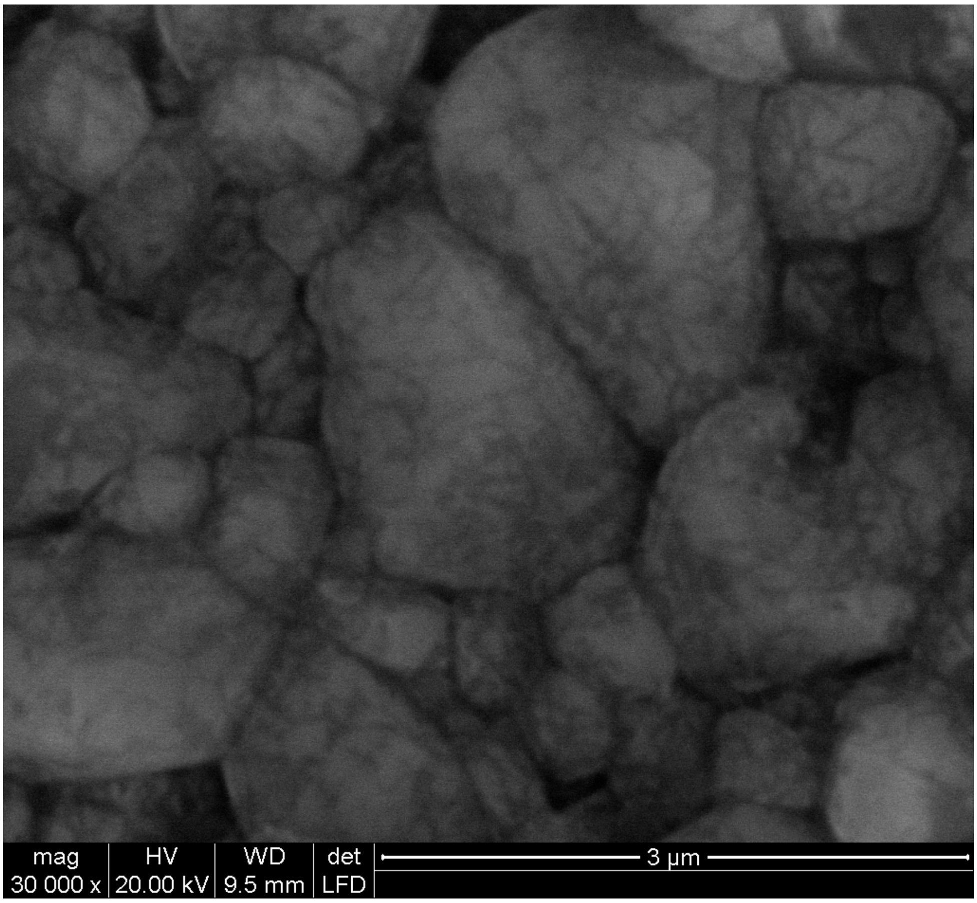

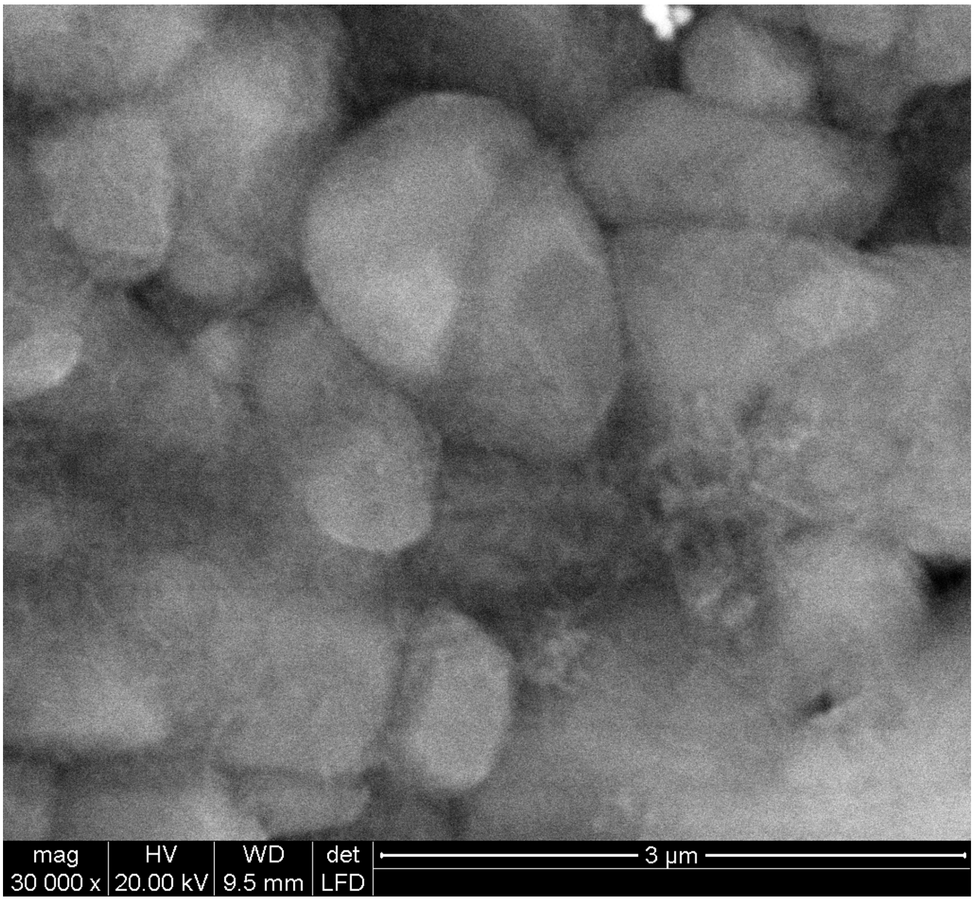

Figure S.2: SEM images of a MWCNT/Au film (top) and of a MWCNT/Au/MHDA film (bottom) deposited on an alumina substrate.

In figure S.2, the grains correspond to the alumina substrates. The MWCNTs can be observed as a very thin mesh on top of the alumina. After functionalization with MHDA, the very thin mesh of MWCNTs is still visible and no significant morphological changes are visible, which indicates that the functionalization is correct and that the subsequent cleaning procedure has prevented the formation of a thick structure of multilayer MHDA.

Table S.1: Effect of the sensor operating temperature (room temperature and 120ºC) on the intensity of the response, the response time and the recovery time of a MWCNT/Au/MHDA sensor (A) and a MWCNT/Au sensor (B) to 20 ppm of ethanol.

	Response intensity		Response time		Recovery time	
Sensor type	A	B	A	B	A	B
R.T.	22.5	6	14	27	7	23
120ºC	6	3	12	23	6	20

For both types of sensors, at 120ºC the intensity of response is dramatically reduced and the dynamics of response become faster. Heating of the sensing films results in desorption of ethanol from the surface of active films, which results in a significant decrease in sensor response. This is indicative of an exothermic adsorption process.

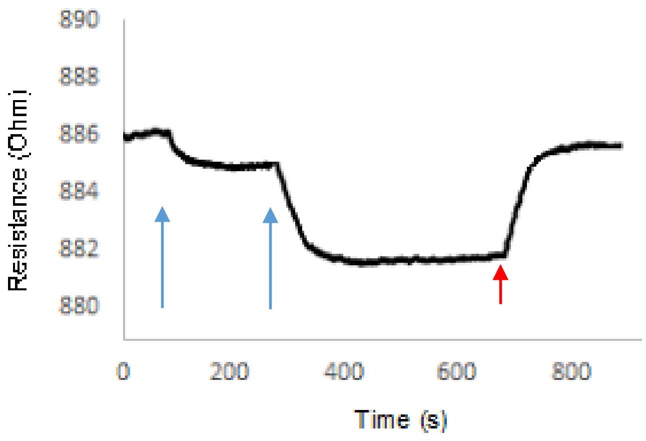

(a)

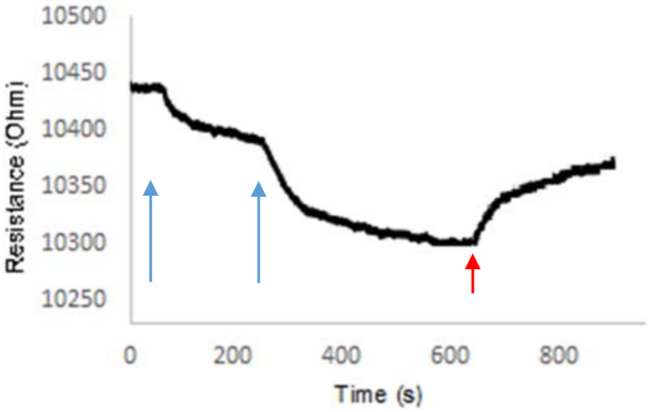

(b)

Figure S.3: Response to NO_2 (100 and 500 ppb) and recovery in dry air of a MWCNT/Au/MHDA sensor (a) and a MWCNT/Au sensor (b). Blue arrows indicate the injection of nitrogen dioxide and the red arrow indicates the injection of pure dry air.

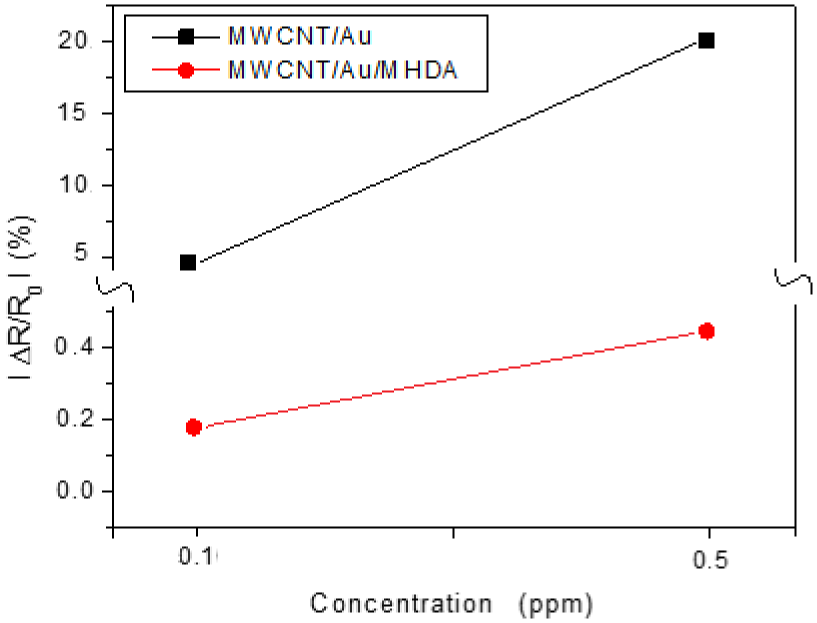

Figure S.4: Calibration curves for the response to NO_2 of MWCNT/Au/MHDA and MWCNT/Au sensors.

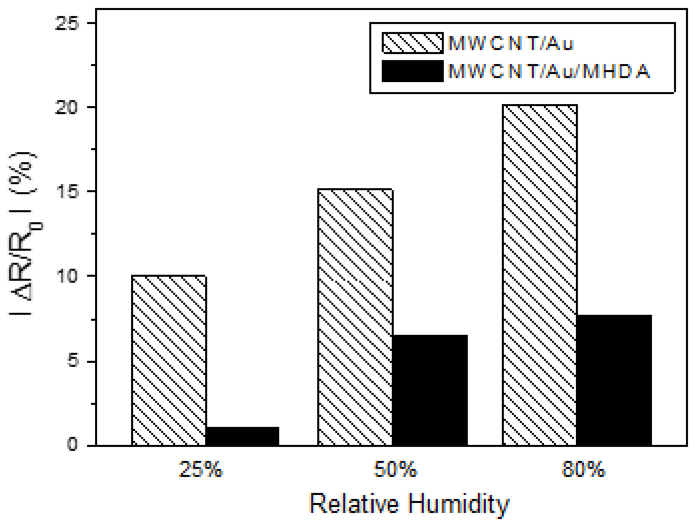

Figure S.5: Response to a sudden change in relative humidity (R.H.) from dry conditions to the R.H. level indicated (@ 25ºC) for MWCNT/Au/MHDA and MWCNT/Au sensors.

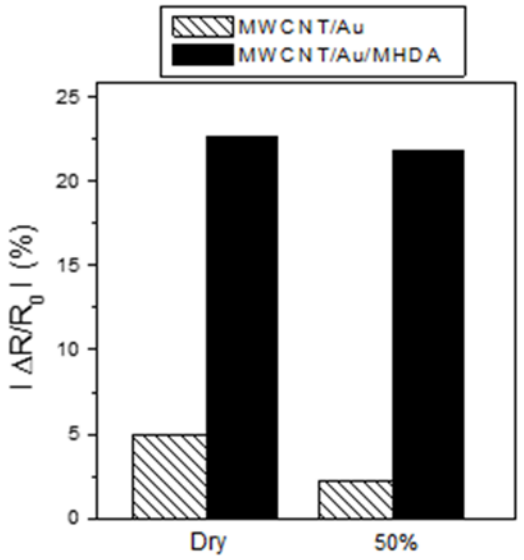

Figure S.6: Response of MWCNT/Au/MHDA and MWCNT/Au sensors to 20 ppm of ethanol under dry or humid (@ 50%, R.H., 25ºC) conditions.
